# Pulsed Low-Dose-Rate Radiotherapy (PLDR): A Comprehensive Review of Radiobiological Rationale and Clinical Applications in Modern Cancer Care

**DOI:** 10.7759/cureus.116488

**Published:** 2026-09-18

**Authors:** James S Welsh, Seyed Alireza Javadinia, Veronia Fahmy, Elham Khakshour, Leili Darvish, Alisher Kahharov, Steven P Howard

**Affiliations:** 1 Radiation Therapy, Edward Hines Jr. Veterans Administration (VA) Hospital, Hines, USA; 2 Radiation Oncology, Loyola University Chicago Stritch School of Medicine, Maywood, USA; 3 Radiation Oncology, Sabzevar University of Medical Sciences, Sabzevar, IRN; 4 Radiation Oncology, Loyola University Medical Center, Maywood, USA; 5 Cellular and Molecular Research Center, Sabzevar University of Medical Sciences, Sabzevar, IRN; 6 Mother and Child Welfare Research Center, Hormozgan University of Medical Sciences, Bandar Abbas, IRN; 7 Radiation Oncology, Tashkent State Dental Institute, Tashkent, UZB; 8 Radiation Oncology, University of Wisconsin School of Medicine and Public Health, Madison, USA

**Keywords:** clinical radiobiology, dna damage response, glioblastoma re-irradiation, low-dose hyper-radiosensitivity, normal tissue sparing, pulsed low-dose-rate radiation therapy, radiotherapy, re-irradiation, sublethal damage repair

## Abstract

Pulsed low-dose-rate radiotherapy (PLDR) has emerged as a promising retreatment strategy that delivers radiation in a series of low-dose pulses, potentially increasing sublethal damage repair in normal tissues while exploiting low-dose hyper-radiosensitivity and impaired DNA repair mechanisms in tumor cells.

This review synthesizes current evidence on PLDR by outlining its historical development, radiobiological foundations, and evolving clinical applications across tumor sites, with particular attention to re-irradiation settings. Preclinical studies suggest enhanced normal-tissue sparing and modulation of cellular responses under pulsed dose-rate conditions, supporting the mechanistic rationale for clinical use. Early clinical experience indicates favorable tolerability and potential therapeutic benefit in brain, spine, head and neck, breast, and pelvic malignancies, although these data are predominantly retrospective and heterogeneous.

Despite these encouraging findings, major challenges remain, including limited prospective data, variation in dose-fractionation schemes, and incomplete understanding of long-term safety. Collectively, available evidence positions PLDR as a feasible and biologically grounded option for patients requiring re-irradiation, while underscoring the urgent need for standardized protocols and well-designed clinical trials to define its optimal use and long-term therapeutic value.

## Introduction and background

Pulsed low-dose-rate radiotherapy (PLDR) is an emerging technique originally introduced to manage large-volume recurrent tumors, particularly in previously irradiated areas where conventional retreatment carries high risks. The radiobiological concept underlying PLDR emerged several decades ago from observations of low-dose hyper-radiosensitivity (HRS) and dose-rate effects, with subsequent development into a clinically applicable re-irradiation strategy through advances in radiation delivery technology [1-4].

The key difference between PLDR and conventional low-dose-rate (LDR) or high-dose-rate (HDR) radiotherapy is the delivery of dose and biological effects. In PLDR, the daily dose is divided into multiple small pulses (<0.2-0.3 Gy) delivered at short intervals, separated by three minutes, which yields a low effective dose rate. In clinical practice, the general target is an effective dose rate of 6.7 cGy/minute (0.067 Gy/minute). This approach takes advantage of tumor cell hypersensitivity to low doses while also allowing normal tissues time to repair sublethal damage, thereby enhancing tumor control and reducing toxicity [5,6]. By contrast, conventional LDR delivers radiation continuously at 0.4-2 Gy/hour without pulse fragmentation, which may permit simultaneous repair in both tumor and normal cells. HDR, on the other hand, administers several Gy per minute in a short burst, causing rapid tumor cell damage but also higher risks of normal tissue injury. In summary, PLDR offers a therapeutic balance between efficacy and safety, making it particularly valuable for re-irradiation in previously treated areas [3,4,7-10].

PLDR offers several biological advantages that enhance efficacy while reducing toxicity. One of the most important is the protection of normal tissues: dividing the total dose into small pulses separated by at least three minutes allows time for repair of sublethal damage, lowering cumulative toxicity compared with HDR delivery [6,11]. The proposed radiobiological rationale for PLDR involves several interrelated mechanisms, including low-dose HRS, differential sublethal damage repair (SLDR) between normal and malignant tissues, and potential effects on tumor oxygenation, cell-cycle distribution, vascular biology, and radiation-induced inflammatory signaling, including a reduction in induction of transforming growth factor β (TGF-β) compared with conventional radiation therapy (RT) [12-14].

This review aims to synthesize the current knowledge on PLDR by outlining its historical background, radiobiological basis, and clinical applications. It also summarizes the practical considerations and technical aspects that shape PLDR delivery in modern radiotherapy. Furthermore, the review highlights key challenges, including the limited number of prospective clinical studies, heterogeneity in treatment parameters, and the incomplete understanding of long-term safety, and discusses future needs such as establishing standardized dose constraints, optimizing pulse-fractionation schemes, and conducting systematic trials to validate the durability and safety of this technique.

## Review

Current understanding of PLDR mechanisms: biological basis

Low-Dose HRS and IRR

Recent experimental data indicate that many tumor cell systems exhibit hypersensitivity to low doses in the range of ~0.1-0.3 Gy, followed by the development of an increased radioresistance plateau as the dose per fraction rises [15]. One principle of PLDR exploits this phenomenon by dividing a fraction into multiple subfractions of 0.3 Gy or less, thereby repeatedly engaging the HRS window within a single treatment fraction. This should theoretically enhance tumor cell kill beyond predictions of the conventional linear-quadratic (LQ) model [1,5]. Mechanistic studies suggest that HRS arises from inadequate activation of DNA damage checkpoints and a delayed onset of error-free repair pathways at very low doses, whereas higher single doses restore checkpoint signaling and induce radioresistance (IRR). Recent clonogenic survival analyses [16] and in vivo chromosome break assays [17] further reinforce the dose- and rate-dependence of HRS/IRR, supporting the clinical rationale for applying PLDR to maximize therapeutic benefit.

DNA Damage/Repair Response

Ionizing radiation generates diverse DNA lesions, but double-strand breaks (DSBs) remain the most cytotoxic and are the key determinant of clonogenic survival. Their recognition initiates the DNA damage response (DDR), coordinated by ataxia telangiectasia mutated (ATM) and ATM and Rad3-related kinases, which propagate signaling through Chk2/Chk1 checkpoints, chromatin modifiers (γH2AX, MDC1), and repair effectors in nonhomologous end joining and homologous recombination (HR) pathways [18,19]. In the context of PLDR, the temporal pattern of delivery modifies DDR dynamics, since both checkpoint activation and repair fidelity are dose- and time-dependent. Unlike conventional high-dose exposures, where ATM activation is robust and checkpoint stabilization prevents cell-cycle progression until repair is complete, very small pulses, as employed in PLDR, may induce only subthreshold DDR activation, resulting in incomplete checkpoint arrest, delayed HR machinery recruitment, and persistence of DSBs that predispose tumor cells toward misrepair or apoptosis. Normal tissues can clear sublethal lesions during interpulse intervals, thereby preserving viability and contributing to PLDR’s therapeutic selectivity [15,20].

Tumor Vasculature Responses to Pulsed Low-Dose Radiation

Endothelial cell death and subsequent vascular collapse are well-recognized consequences of radiation, but the dose-rate and fractionation scheme markedly influence both [21]. Based on the study by Dilworth et al. [22], pulsed low-dose irradiation of orthotopic glioblastoma multiforme (GBM) in a preclinical model (mimicking the clinical PLDR technique of ten 0.2-Gy pulses separated by three-minute intervals) demonstrated significant effects on vascularization and tumor control. The study found that PLDR resulted in improved tumor control and survival compared with conventional HDR regimens using daily 2-Gy fractions, with a statistically significant (p = 0.05) 28% reduction in degenerating neurons in the surrounding brain parenchyma. This improvement was attributed to enhanced vascularization and preservation of the tumor microenvironment, which may contribute to reduced hypoxia-driven radioresistance and increased tumor radiosensitivity [22]. Preclinical findings suggest that PLDR may preserve aspects of vascular integrity and perfusion in some treatment contexts; however, radiation-induced endothelial injury and vascular dysfunction may also contribute to tumor response. Thus, vascular effects of PLDR may be context-dependent and could represent both a potential normal-tissue-sparing mechanism and a contributor to tumor control.

The result appears to be a reduction in hypoxia-driven radioresistance and improved tumor radiosensitivity. In vivo preclinical work corroborates this hypothesis of vascular sparing. Mouse models receiving PLDR showed significantly better preservation of microvascular integrity and blood flow within target tissues compared with standard continuous delivery, with associated reductions in normal tissue toxicity [23].

Radiobiological Modeling

Traditional radiobiological models, including the LQ model, have long served as the foundation for predicting cellular responses to radiation; however, they exhibit several limitations when applied to PLDR. The classical LQ model assumes continuous and homogeneous dose delivery, thereby failing to account for biological effects arising from interpulse repair kinetics or for low-dose phenomena such as HRS and induced IRR, which are central to the therapeutic rationale of PLDR. Modern modeling methods, therefore, incorporate sublethal damage repair kinetics, for example, via the Thames “g-factor” or explicit repair-rate equations, to calculate a more accurate biologically effective dose [24,25], taking into account both pulse size and timing. Consensus guidance (e.g., AAPM TG-267) emphasizes the need to include repair half-times (T₁/₂), often 10s of minutes to several hours, when modeling PLDR schedules [26]. Experimental evidence supports this paradigm shift. In vitro experiments showed that survival outcomes depend not only on total dose but also on the delivery structure: the same fractional dose delivered as pulses with defined gaps can yield different biological outcomes [25]. In vivo murine experiments further showed that multiple small pulses (e.g., 20-30 cGy every few minutes) yield lower normal-tissue toxicity than a continuous dose, aligning well with repair-aware scheduling approaches [23]. By capturing these time-dependent effects, contemporary models provide a more realistic estimation of therapeutic efficacy and normal-tissue sparing, advancing the precision of PLDR treatment planning.

A simplified repair-aware model considers the interaction between dose per pulse, interval between pulses, and the kinetics of SLDR. When two radiation exposures are separated by an interval t, the residual unrepaired damage can be approximated using an exponential repair model based on a characteristic repair half-time. The resulting biological effect therefore depends not only on total dose but also on pulse size and temporal spacing. More sophisticated formalisms incorporate incomplete repair using Lea-Catcheside-type factors or related repair models.

*SLDR* *Dynamics Between Pulses*

A distinctive radiobiological feature of PLDR is its ability to exploit the differential kinetics of SLDR between normal and tumor cells. While conventional fractionated radiotherapy delivers a continuous dose (usually 2 Gy per daily fraction over about a minute) in which repair and damage overlap, PLDR deliberately introduces short interpulse intervals, typically three minutes, allowing partial DNA damage resolution in normal tissues before the next pulse. This interval is particularly relevant because the effective repair half-times (T½) of many mammalian tissues fall in the range of 15-40 minutes, meaning that even modest breaks between pulses provide biologically meaningful opportunities for repair [6,13,14]. Recent evidence highlights that SLDR between radiation pulses is not solely governed by classical DNA repair kinetics but is also influenced by molecular regulators such as the 53BP1/RIF1 signaling axis, which promotes cell survival under multifractionated regimens. However, persistent exposure to fractionated low-dose radiation can induce the accumulation of DNA damage and trigger profound alterations in DNA and histone methylation, particularly within sensitive tissues such as the thymus. These epigenetic changes, coupled with incomplete repair processes, may compromise genomic stability and alter long-term cellular fate. Together, these findings underscore the dual nature of SLDR dynamics, where efficient repair pathways can sustain cell viability in the short term, but cumulative genetic and epigenetic perturbations may contribute to delayed radiosensitivity or adaptive resistance [27,28].

Reducing Normal Tissue Toxicity

One of the defining advantages of PLDR is its ability to minimize normal tissue toxicity compared with conventional radiotherapy (CRT) delivered at equivalent doses. Preclinical animal studies have consistently demonstrated that PLDR increases tolerance thresholds in critical organs, resulting in improvements in survival and reductions in systemic toxicity. For instance, murine models of whole-abdominal irradiation demonstrated that the lethal dose (LD₅₀) for PLDR was approximately 29 Gy (more than 60% higher than CRT). PLDR was also associated with less body weight loss, prolonged survival, and reduced histopathological tissue damage, such as intestinal atrophy and hyperplasia [7]. Consistent findings were reported in other in vivo animal models, showing that genotoxicity and inflammatory markers, including comet assay parameters (tail DNA%) and TGF-β expression in bone marrow and gut tissues, were significantly more pronounced in CRT than in PLDR groups (p < 0.001 for multiple comparisons). These data highlight the superior protective effect of PLDR against acute and subacute normal tissue injury [23]. Additional support for this tissue-sparing effect comes from tumor-bearing models, where PLDR preserved vascular integrity and perfusion in peritumoral regions while reducing normal brain tissue damage in orthotopic GBM models. Preserving microvascular function was shown to limit hypoxia-driven radioresistance and simultaneously improve tumor radiosensitivity [22]. These findings establish PLDR as a regimen capable of expanding the therapeutic window by maintaining tumor control while significantly reducing acute and subacute toxicity in vital normal tissues. This dual advantage provides a strong rationale for its translation into clinical reirradiation settings, where cumulative normal tissue dose constraints often limit conventional regimens [13].

Preclinical evidence

Animal Models

Animal models have been instrumental in unmasking the biological and therapeutic impacts of PLDR in different kinds of tumors. For glioblastoma models, PLDR substantially delays tumor growth and prolongs survival compared with CRT, partly through improved blood vessel preservation and reduced tumor radioresistance due to hypoxia [22]. In prostate cancer models, pulsed delivery of low-dose radiation resulted in greater tumor radiosensitivity than continuous irradiation and was accompanied by delayed growth and elevated apoptotic indices [29]. Importantly, murine lung and abdominal model studies have confirmed the cytoprotective action of PLDR on normal tissues: threshold lethal doses were significantly higher in PLDR-treated groups than in regular regimens, with reduced weight loss, less gastrointestinal toxicity, and improved histopathological findings [7].

Moreover, studies in murine lung, abdominal, and pelvic models, including work by Wen et al. [1], have demonstrated that PLDR improves tumor control while simultaneously sparing normal tissues. In these models, PLDR-treated animals tolerated higher cumulative doses, with reduced weight loss, decreased gastrointestinal toxicity, and improved histopathological outcomes compared with conventional regimens. These findings confirm the translational value of PLDR for pelvic, abdominal, and other solid malignancies, highlighting its dual advantage of facilitating tumor control with decreased normal tissue damage [1]. More recent evidence from liver cancer models also confirms these findings. In a hepatocellular carcinoma rat model, PLDR effectively inhibited tumor growth without damaging normal hepatic tissues, as indicated by reduced serum alpha-fetoprotein (AFP) and interleukin-6 (IL-6) levels, better weight maintenance, and fewer histologic abnormalities than with CRT [30]. These animal data collectively hint at a preservation of tumor control with a simultaneous reduction in normal tissue complications in most organ systems.

PLDR vs. Conventional RT Outcomes: Tumor Control, Toxicity, and Immune Response

Evidence from both preclinical and clinical studies indicates that PLDR achieves comparable or even superior tumor control relative to CRT, with markedly reduced normal tissue toxicity. In glioblastoma models, PLDR reduced tumor growth by more than 50% compared with conventional irradiation, translating into median survival advantages of approximately 20%-30% in irradiated mice [22]. In a mouse model, pulsed LDR radiation therapy (PLRT) plus temozolomide produced greater tumor growth delay and less normal brain damage than conventional RT plus temozolomide [31]. Normal tissue sparing is one of the most consistent advantages of PLDR across animal studies. In murine whole-abdominal irradiation models, the 50% lethal dose of radiation (LD₅₀) was approximately 29 Gy in PLDR (more than 60% higher than with single-fraction irradiation), with decreased weight loss, decreased gastrointestinal toxicity, and improved histopathological scores [7]. Similar protective effects were observed in lung irradiation studies, where PLDR groups showed lower rates of pulmonary inflammation and fibrosis than conventionally irradiated Wistar rats [23]. These findings support the idea that PLDR can expand the therapeutic window by maintaining tumor control, while significantly reducing normal tissue complications. Clinical experiences generally substantiate these findings. In a cohort of 13 patients with recurrent head and neck, lung, pelvic, and uterine/cervical cancers re-irradiated with PLDR, Tong et al. published three complete responses and 10 partial responses, with acute and late toxicities limited to grade 1-2 in most of the patients [32]. Similarly, a pending multicenter phase II trial is underway to test PLDR in recurrent esophageal squamous cell carcinoma, which is an indicator of growing confidence in its clinical utility [33].

A growing body of preclinical evidence suggests that the immune-modulatory effects of PLDR contribute significantly to its therapeutic selectivity. In particular, animal studies comparing PLDR with CRT have shown consistent differences in inflammatory and fibrotic signaling pathways. In a recent in vivo study, toxicity markers in bone marrow and gut tissues demonstrated that PLDR animals exhibited significantly lower levels of genotoxic stress and inflammatory responses. Specifically, comet assay parameters (tail DNA%) were reduced, and the elevation of the profibrotic cytokine TGF-beta was reduced with PLDR compared with conventionally treated groups, underscoring the ability of PLDR to suppress tissue-damaging immune cascades [12,23]. Similarly, examination of tissue TGF-beta expression profiles revealed that PLDR results in diminished TGF-beta upregulation in multiple organs compared with conventional radiation, suggesting less activation of the profibrotic and immunosuppressive pathways that drive late radiation injury [7]. These findings suggest that, beyond direct cytotoxicity, PLDR exhibits a favorable immune-modulatory profile by mitigating profibrotic TGF-β signaling and inflammatory stress responses. This immunological advantage likely contributes to the reduced late normal tissue toxicity and may also facilitate more effective antitumor immunity by limiting stromal immunosuppression. Interestingly, it has been speculated that the favorable immunological effects of PLDR could enhance the efficacy of cancer immunotherapy with checkpoint inhibitors. By reducing TGF-beta, PLDR might synergize better with immunotherapy than conventional fractionated RT does [34,35].

Dosimetric and technical considerations

Pulsed Doses

The “pulsed doses” in PLRT (also called pulsed reduced dose-rate radiation therapy or PRDR) mean dividing the total daily radiation dose into several smaller sub-doses, or sequential “pulses.” Each pulse has a specific dose separated from other pulses by specific time intervals (typically three minutes), so each pulse stays below the tumor sensitivity threshold (transition dose). The transition dose (or Dc) in low-dose HRS is the dose threshold beyond which tumor cell radiosensitivity shifts from a highly sensitive state to increased radioresistance. Dc typically varies between 0.2 and 0.6 Gy (or 20-60 cGy), depending on the specific cell type and genotype (e.g., TP53 status). For this reason, most pulses are given in the 20-30 cGy range. This approach is designed so that cancer cells show HRS to this low dose and are more vulnerable, while normal tissues have the opportunity to repair the damage in the intervals between pulses. For example, dividing the daily dose of 2 Gy into 10 pulses with an interval of three minutes provides an effective dose rate of about 0.067 Gy/minute, which increases the effectiveness of radiation on cancer cells and reduces the toxicity of normal tissue [36,37]. The advantage of this approach in the treatment of resistant cancers and recurrent tumors is well-established, as small pulsed doses provide a controlled, fractionated delivery of radiation. Technically, pulsed doses can be delivered using standard linear accelerators and advanced techniques such as intensity-modulated radiation therapy (IMRT) and volumetric-modulated arc therapy (VMAT), which maintain high dose control and precision [38].

Delivery Methods (IMRT, VMAT, 3DCRT, and Proton Beam Therapy)

Although PLDR was originally implemented using 3D conformal radiation therapy (3DCRT), technical implementation of PLDR has been facilitated by modern external beam delivery platforms, particularly IMRT, VMAT, and helical tomotherapy. These methods provide the flexibility to subdivide standard fraction sizes into multiple low-dose pulses, while maintaining conformal target coverage and sparing of normal tissues. Among these, IMRT and VMAT have been studied most extensively. Dosimetric investigations have shown that both IMRT and VMAT can be adapted for pulsed delivery without compromising conformity or organ-at-risk sparing. For example, Lin et al. demonstrated the feasibility of PLDR using IMRT and VMAT for re-irradiation, showing accurate dose reconstruction and clinically acceptable delivery times [39]. By modulating the intensity of individual beamlets, IMRT enables highly conformal dose distributions while segmenting the total fraction into multiple sub-0.3 Gy pulses. Lin et al. demonstrated that IMRT can be adapted for PLDR re-irradiation, achieving accurate delivery of pulse sequences with clinically acceptable treatment times in recurrent head and neck and lung cancers [39]. Clinical data indicate that PLDR-IMRT is feasible and well tolerated for re-irradiation of recurrent nasopharyngeal carcinoma (NPC), with favorable outcomes in tumor control and limited toxicity, as shown in a prospective multicenter phase II trial [40]. For central nervous system (CNS) tumors, evidence remains more limited, but institutional series using PRDR strategies, delivered via IMRT, have reported that re-irradiation is both technically feasible and associated with acceptable toxicity [41].

VMAT-based PLDR provides additional advantages by enabling pulsed delivery during continuous gantry rotation. Ma et al. showed that dynamic arc-based PLDR delivery can maintain consistent pulse fractionation with high temporal accuracy while reducing treatment complexity compared with the static-field IMRT approach [42]. Tyagi et al. reported that VMAT can implement PLDR with high temporal accuracy while reducing treatment time compared with fixed-beam IMRT. This dynamic arc-based approach simplifies delivery complexity and maintains precise pulse structure, offering improved patient comfort and reduced intrafraction motion errors [43]. Importantly, VMAT’s ability to deliver pulses with high temporal resolution has been highlighted as critical for exploiting the low-dose hypersensitivity window. By contrast, 3DCRT represents an earlier generation of conformal techniques that has been applied in some exploratory PLDR contexts. While feasible for dividing total fractions into subdoses, its reliance on fixed beam arrangements limits conformality and results in higher normal tissue exposure compared with IMRT and VMAT. Consequently, 3DCRT-based PLDR is now largely of historical interest, as modern approaches have shifted toward more advanced modalities better suited to exploit the radiobiological advantages of pulsed delivery [13]. These studies establish IMRT and VMAT as the most practical and effective methods for PLDR delivery, combining accurate dosimetry with clinical feasibility, while 3DCRT provides historical proof of principle but limited precision for complex cases.

In addition to these techniques employing conventional linear accelerators, PLDR using helical tomotherapy has been developed and implemented clinically [44,45]. Furthermore, investigations of PLDR using proton beam therapy are currently underway. In this context, the PLDR technique is known as “temporally modulated pulsed proton re-irradiation” or TMPPR. The TMPPR technique delivers a maximum pulse dose of 0.3 Gy/fraction to the clinical target volume separated by five-minute intervals to give an overall time-averaged effective dose rate of < 7 cGy/minute [46].

*Machine Limitations and QA Considerations in PLDR Deliver*y

Implementing PLDR poses challenges for radiotherapy machines because of extended treatment times and the need for precise dosimetric and positional accuracy. Dividing a fraction into multiple small pulses with interpulse intervals prolongs both the beam-on and overall session durations. Planning and delivery studies report that pulsed VMAT/IMRT schedules increase per-fraction delivery time relative to a single continuous fraction due to repeated arc/field passes and intentional interpulse delays [39]. Additional time from gantry rotation, multileaf collimator (MLC) repositioning, and per-pulse verification amplifies these delays, raising concerns about patient comfort, intrafraction motion, and departmental throughput. Accurate dose delivery is also critical: low instantaneous dose rates, MLC leaf-width motion errors, gantry sag or drift, and dose linearity at low MU settings can compromise pulse fidelity, particularly in VMAT-based PLDR, where continuous dose modulation adds complexity. Jagadeesan and Chamundeeswari measured average dose rates of ~285.8 ± 139.07 MU/minute during pulsed VMAT, significantly below the maximal machine rate (600 MU/minute), highlighting limitations in delivering small MU pulses accurately [47]. Dosimetric quality assurance (QA) assessments using delivery logs and electronic portal imaging device analyses show that modern linacs can achieve high accuracy, but rapid leaf or dose-rate changes produce nontrivial deviations. Machine-specific limits, which include minimum MU per segment, dose-rate granularity, and MLC speed/accuracy, determine practical pulse sizes and interpulse timing, making rigorous QA, low-dose calibration, pulse-by-pulse verification, and workflow optimization essential. Integrating automated checks, real-time dosimetry, and advanced planning algorithms helps bridge the gap between theoretical radiobiologic promise and practical delivery.

Clinical evidence and ongoing trials

Clinical application of PLRT has expanded over the past decade, primarily in the re-irradiation of large-volume recurrent and radioresistant tumors. Early institutional experiences demonstrated encouraging tumor control rates and low toxicity, supporting its translation from preclinical models to patient care.

Head and Neck and CNS Tumors

Recurrent head and neck cancers have been among the most common indications for PLDR due to the proximity of radiosensitive structures and cumulative dose limitations. In a prospective multicenter trial, Huang et al. [40] evaluated PLDR-IMRT in recurrent NPC and reported a one-year local control rate of 78% with no grade ≥3 late toxicities, confirming both feasibility and tolerability. Moreover, Kutuk et al. demonstrated that PLDR IMRT for recurrent CNS malignancies achieved long-term local control with a median overall survival of over 12 months and acceptable neurotoxicity [41]. Collectively, these studies suggest that PLDR may overcome retreatment barriers in heavily preirradiated regions while maintaining functional tissue preservation.

Thoracic and Abdominal Malignancies

PLDR has also shown promise in thoracic and abdominal cancers, including lung, liver, and gastrointestinal tumors. Wen et al. reported improved therapeutic efficacy of PLDR in abdominal and pelvic malignancies, with reduced intestinal and hepatic toxicity compared with conventional re-irradiation [1]. In a rat model of hepatocellular carcinoma, Salem et al. showed that PLDR achieved tumor control comparable to conventional RT but with lower IL-6 and AFP levels, confirming hepatoprotection [30]. Clinically, Tong et al. applied PLDR to patients with recurrent thoracic and pelvic tumors and achieved an overall response rate of 100% (three complete, 10 partial responses) with only grade 1-2 toxicities [32]. These findings highlight PLDR as a rational option for re-irradiation where dose constraints traditionally preclude effective treatment.

Gliomas and Other CNS Tumors

The PLDR technique was initially developed to treat recurrent gliomas too large to undergo retreatment with stereotactic radiosurgery [4]. This study from the University of Wisconsin addressed recurrent gliomas (mostly GBM) with a mean treatment volume of 403.5 cc based on T2-weighted MRI and a 2-cm margin. This contrasts with a median treatment volume in older series of <74 cc. Despite this large volume and cumulative doses >100 Gy, PRDR allowed safe retreatment with palliative benefit that was well tolerated overall. When PLDR was given for recurrences 14 months or more after initial radiotherapy, median survival was significantly improved compared with retreatment given for recurrent disease less than 14 months after initial treatment. In another study from the University of Wisconsin, 58 patients with recurrent grade 2 gliomas received PRDR. Overall survival after PRDR was 12.6 months. Among patients who maintained grade 2 histology at recurrence (31%), overall survival was 22.0 months, with five patients remaining disease-free long term; the longest survivors were 8.2 and 11.4 years at the time of publication. Again, PRDR was generally well tolerated with acceptable toxicity profiles [48].

In a study from the Cleveland Clinic, 24 patients received PRDR for recurrent brain tumors between 2012 and 2014 [49]. The median PRDR dose was 54 Gy (range, 38-60 Gy), and 50% of the patients had ≥4 chemotherapy regimens before PRDR. The target volumes were large, with a mean planning target volume of 369.1 ± 177.9 cc. The median overall survival was 8.7 months, and the six-month survival rate was 71%. The authors note that toxicity was similar to what was seen in initial treatments, with no cases of radiation necrosis noted. In an update from the Cleveland Clinic team, 55 patients with recurrent primary CNS tumors and a mean number of salvage treatments before PRDR of 2.83 were re-treated with a median dose of PRDR of 54 Gy in 30 fractions. The mean dose of prior RT was 57.75 Gy, bringing the cumulative dose well over 100 Gy. The observed overall survival was 10.98 months, and treatment was well tolerated, with 86.6% of the adverse events being grade 1-2. There were three grade 3 adverse events and three grade 4 side effects, but these were all attributed to the concurrent chemotherapy; there was no radiation necrosis observed. The authors concluded that PLDR is a safe and effective re-treatment approach for heavily pretreated patients with recurrent primary CNS malignancies.

In a study of PLDR for eight patients with recurrent meningioma, six had histologically confirmed atypical meningiomas. The median time between radiation courses was 7.7 years, and the median PRDR dose was 54 Gy in 27 fractions to a median volume of 261.6 cc. Progression-free survival at six months was 100%. On longer follow-up, two patients (25%) had in-field failures, with a median follow-up of 23.3 months. A total of 87.5% of the patients were still alive at the time of the publication, and none had grade ≥ 2 acute or late toxicities [10].

PLDR has also been used as a method of re-treating patients who had prior whole-brain RT and needed additional therapy. Seventy-five patients received repeat whole-brain RT using PRDR with a median dose of 26 Gy in 2 Gy fractions. Of these 75 patients, 32 patients had over 10 brain metastases, and 11 had leptomeningeal disease. The median overall survival was 4.1 months (range, 0.29-59.5 months). The authors concluded that PRDR confers acceptable survival and may decrease toxicity associated with repeat whole-brain RT for multiply recurrent brain metastases [9].

In the context of CNS re-irradiation, Prabhu et al. provide one of the few documented clinical experiences involving spinal cord retreatment with PRDR. The paper describes two patients with recurrent intracranial and spinal tumors treated safely within previously irradiated fields. Notably, one case involved a thoracic intramedullary glioblastoma re-irradiated using 50 Gy delivered in 250 pulses over six weeks, resulting in neurological improvement, specifically, regained ability to ambulate with a walker, without evidence of radiation-induced myelopathy [50]. Although the sample size is extremely limited, these observations highlight the potential radioprotective advantages of a pulsed LDR schedule in the spinal cord, a tissue with minimal tolerance to cumulative dose. Including this evidence emphasizes the expanding applicability of PLDR in highly dose-constrained CNS regions and underscores the need for more systematic investigation to characterize its long-term safety and therapeutic durability.

Although PLDR has primarily been investigated as a re-irradiation strategy, prospective clinical data also exist in the upfront setting. Almahariq et al. evaluated PLDR in patients with newly diagnosed glioblastoma and incorporated longitudinal assessments of neurocognitive function and quality of life [51]. The study demonstrated the feasibility of PLDR in the initial treatment setting, with reported preservation of neurocognitive function and quality of life during follow-up. These findings provide preliminary support for further investigation of PLDR outside the re-irradiation setting but do not establish superiority over standard chemoradiotherapy.

Despite a compelling radiobiological rationale, PLDR has not replaced conventional fractionation as the standard approach for newly diagnosed malignancies. Several factors may explain this. First, the principal clinical rationale for PLDR has historically been the need to reduce cumulative normal-tissue injury in previously irradiated patients, where conventional retreatment may be limited by prior dose exposure. This therapeutic problem is less prominent during initial treatment, for which established fractionation schedules have extensive prospective evidence supporting tumor control and long-term toxicity outcomes. Second, PLDR requires prolonged treatment times and specialized delivery workflows, potentially increasing treatment-room occupancy and intrafraction motion. Third, the proposed biological advantages of PLDR, including low-dose HRS and differential SLDR, may not be uniform across tumor types and molecular contexts. Finally, PLDR has not yet been demonstrated in adequately powered randomized trials to provide superior tumor control or reduced toxicity compared with contemporary standard therapy. Accordingly, the strongest current rationale for PLDR remains in carefully selected re-irradiation settings, while upfront applications should be considered investigational.

The same principle applies to other advanced radiation approaches, including proton therapy and brachytherapy: a theoretical dosimetric or biological advantage does not automatically establish clinical superiority. Prospective comparative trials are necessary to determine which patients derive meaningful benefit.

Multicenter phase II/III trials and future perspectives

Additional clinical trials have been proposed and started to determine the safety and efficacy of PLDR for various sites of recurrent disease and for use as an up-front initial treatment. An example is the multicenter phase II trial by Li et al. on PLDR in locally recurrent esophageal squamous cell carcinoma after prior radiotherapy [33]. Early interim reports indicate that PLDR achieves meaningful tumor regression with limited esophageal stricture and mucosal toxicity. Furthermore, clinical feasibility studies using VMAT- and IMRT-based PLDR platforms [39,42] have validated the reproducibility of pulse delivery and dose homogeneity, paving the way for protocol standardization.

One interesting upfront trial using PLDR is a phase II study called the Pulsed Reduced Dose Radiotherapy in Upfront Glioblastoma study. This is a single-arm, single-center, phase 2 study through the Medical College of Wisconsin, substituting PLDR for standard RT. It is designed to assess the efficacy of pulsed reduced dose-rate radiotherapy as initial treatment for maximally safely resected glioblastoma and will test the hypothesis that PLRT may be more effective than conventional RT in certain circumstances. As clinical evidence accumulates, PLDR is emerging as a potential standard salvage therapy for recurrent or radioresistant disease and warrants inclusion in future consensus guidelines.

More recently, NRG-CC017 (NCT07452458) was initiated as a randomized study evaluating PLDR in newly diagnosed glioblastoma. This trial will provide important prospective comparative data to determine whether the proposed radiobiological advantages of PLDR translate into improved clinical outcomes compared with CRT.

## Conclusions

PLDR represents an innovative adaptation of conventional radiation delivery that leverages intrinsic radiobiological vulnerabilities in tumor cells while potentially sparing normal tissues from excessive cumulative damage.

PLDR remains an investigational salvage strategy with encouraging early clinical experience, but available evidence is insufficient to establish it as a standard treatment. However, the evidence remains heterogeneous, with few prospective studies and limited long-term follow-up. Moving forward, systematic clinical trials, clearer dose-fractionation standards, and rigorous evaluation of long-term safety are essential to defining the true therapeutic value of PLDR. Despite current knowledge gaps, the expanding clinical interest in PLDR underscores its promise as a meaningful option for patients with limited retreatment alternatives.
